# Current Approaches to Four Challenging Pain Syndromes

**DOI:** 10.7759/cureus.45573

**Published:** 2023-09-19

**Authors:** Eleni Moka, Abdallah El-Sayed Allam, Martina Rekatsina, Lynda Abed, Antonella Paladini, Abdullah AlKharabsheh, Athina Vadalouca, Giustino Varrassi

**Affiliations:** 1 Department of Anesthesiology, Creta InterClinic Hospital, Herakleion, GRC; 2 Morphological Madrid Research Center (MoMaRC), UltraDissection Spain EchoTraining School, Madrid, ESP; 3 Physical Medicine, Rheumatology and Rehabilitation, Tanta University Hospitals & Faculty of Medicine, Tanta University, Tanta, EGY; 4 Pain Management, Basildon University Hospital, London, GBR; 5 Anesthesia and Intensive Care, Djilali Bounaama Douera Hospital University, Algiers, DZA; 6 Department of Life, Health and Environmental Sciences (MESVA, University of L'Aquila, L'Aquila, ITA; 7 King Abdullah University Hospital, Jordan University of Science and Technology, Amman, JOR; 8 Pain and Palliative Care Center, Athens Medical Hospital, Athens, GRC; 9 Pain Medicine, Paolo Procacci Foundation, Rome, ITA

**Keywords:** persistent knee pain, knee pain, facet joint pain, chest wall pain, chest pain, arthropathy, chronic postsurgical pain

## Abstract

During a conference of pain specialists, some of the experts addressed the potential management of four prevalent but difficult painful conditions, namely, chronic postsurgical pain (CPSP), knee osteoarthritis, chest trauma, and facet joint arthropathy. In all cases, the conditions posed challenges in accurate diagnoses as well as safe, effective treatments, especially using locoregional blocks. It is not clear why some surgical patients develop CPSP and others do not, although some risk factors have been identified. More importantly, the transitional phase of pain from acute to chronic deserves greater scrutiny. It appears as if more aggressive and more effective perioperative and postoperative analgesia could help mitigate or possibly prevent CPSP. Knee osteoarthritis is prevalent but is often managed pharmacologically and then with joint replacement; many patients simply live with the condition which can be viewed as a disease of the entire joint. New approaches with intra-articular injections of hyaluronic acid, platelet-rich plasma, and botulinum toxin may provide safe, effective, and durable pain control. Chest trauma can be extremely painful and a source of morbidity, but its management tends to rely on watchful waiting and drug therapy. New approaches to regional nerve blocks can be beneficial and may reduce troublesome symptoms such as the inability to cough or clear the lungs. Facet joint arthropathy is very prevalent among older people but is not completely clarified. It may be the source of intense pain with limited management strategies. The role of nerve blocks in facet joint arthropathy is an important new addition to the armamentarium of pain management, particularly for geriatric patients.

## Introduction and background

The oldest challenge in medicine remains the most elusive: understanding pain so that it can be more accurately diagnosed, more effectively treated, and more safely managed. Even the definitions of pain are in a state of evolution and updating [1]. Pain is both adaptive and maladaptive, even destructive. Whether or not chronic pain is a disease unto itself is disputed [2]. Despite advances in pharmacotherapy with new drug targets and technological innovations in deep brain stimulation and neuromodulation, clinicians are still confronted daily with patients who have painful conditions that can be challenging to manage. Many painful conditions common in real-world clinical practice may be underestimated, under-reported, misunderstood, and, as a result, inadequately treated. A recent conference on pain in Tunis, Tunisia, discussed four particular types of pain that are both familiar to general practitioners, emergency clinicians, and pain specialists which deserve a new look in light of our growing elucidation of pain mechanisms and new treatment options.

The four topics selected were chronic postsurgical pain (CPSP), thoracic trauma, facet-joint arthropathy, and knee osteoarthritis. These conditions are prevalent, challenging to manage, and not widely understood. The aim of this series of presentations was not to present an exhaustive list of pain medicine innovations or to describe all of the prevalent but challenging conditions clinicians face each day, but to outline how innovation and elucidation have converged to provide new insights into four old problems.

## Review

Methodology

This paper is based on four presentations given at an international pain conference in Tunis, Tunisia, from May 11 to May 13, 2023. This is a summation of academic presentations.

Results

Chronic Postsurgical Pain

CPSP is emerging as a silent epidemic. While investigators have examined postsurgical pain following specific types of surgical procedures, such as post-thoracotomy pain [3], there remains a paucity of high-quality research on the overall subject and mechanisms behind enduring pain in the aftermath of surgical intervention. There is no universally accepted expert definition of CPSP, although an early and often used definition describes CPSP as pain, emerging after a surgical procedure and that has persisted for ≥2 months when all other causes can be excluded [3]. Later sources maintain that CPSP must have lasted at least three months [4]. Indeed, the definition of CPSP is being scrutinized for an appropriate and accurate definition [4] (Table 1).

**Table 1:** The International Association for the Study of Pain Task Force on Chronic Pain has proposed a new definition for chronic postsurgical pain.

New definition	Changes versus earlier definitions	Comments
Pain developed after a surgical procedure or increased pain intensity after the surgical procedure	Addition of pain that preceded surgery but worsened after surgery	This includes both new and worsened pain
Pain should be of at least three months duration with a significant effect on the quality of life	The original definition was two months Added that this pain must adversely affect the quality of life	This would exclude prolonged pain syndromes that do not affect the quality of life
Pain is a continuation of acute postoperative pain or may develop after an asymptomatic period	Better recognition that CPSP may be preceded by a short postsurgical period without pain	This suggests that, at least in some cases, CPSP may not be a transition from acute postsurgical pain
Pain is localized to the surgical field or to a referred area (e.g., innervation territory, referred dermatome for visceral surgery)	This adds that pain must be localized or referred to the surgical area	
Other possible causes for the pain have been ruled out	This has always been part of the CPSP definition	Other causes for pain following surgery may be infection or recurrence of cancer

One reason for the lack of research into CPSP may be the misconception that postsurgical pain indicates failed surgery or an improper technique. There is also a misconception on the part of patients who may not be aware that CPSP is a potential side effect of any surgical intervention [5]. As CPSP is not widely recognized, it often goes untreated. In fact, CPSP was not listed in the International Classification of Diseases (ICD) until the ICD-11 appeared [6,7].

CPSP is not rare. An observational multicenter study of 3,120 surgical patients reported that moderate to severe CPSP was present in 11.8% of patients at 12 months and 35.4% of patients had neuropathic pain [8]. In that study, it was found that 2.2% of patients had persistent severe pain at one year [8]. A secondary analysis of a prospective study of 14,831 patients ≥45 years who underwent major noncardiac surgery reported that 3.3% had persistent incisional pain at one year and 81% of patients had neuropathic pain features [9]. Overall, 1% of surgical patients experience CPSP so severe it adversely affects their recovery and rehabilitation [8].

Despite progress with new surgical techniques, robotic surgery, laparoscopic procedures, greater understanding of pain mechanisms, analgesics, rehabilitation, and evidence-based pain care, the prevalence of CPSP has remained relatively unchanged [10]. Changes have occurred in our understanding of CPSP, namely, that it can diminish over time and that it sometimes does not occur immediately following surgery but rather occurs after a short asymptomatic period [10]. At least a third of CPSP patients have a neuropathic component to their pain [10].

Risk factors for CPSP have been developed, including the intensity of preoperative pain and the percentage of time a patient experiences severe pain in the first 24 hours after surgery. Other risk factors include emotional stress, preoperative pain at the site of the surgery, severe acute postoperative pain, disordered sleep, and any chronic pain syndrome that existed before surgery, such as migraine or low back pain [10]. Thus, predictive factors can be grouped by those that occur before surgery, such as pre-existing painful conditions and stress [11], factors that occur in the perioperative and immediate acute postoperative phase, such as prolonged experience of severe pain after surgery [8,11], but less is known about the subacute period following surgery. In general, clinical risk factors had the strongest predictive value, while genetics, social status, type of anesthesia (general or regional), adjuvant treatments, and demographics, such as female sex and higher body mass index, were less robust predictors [10,12].

It has long been speculated that CPSP is acute pain that becomes centralized. Certain forms of acute pain may transition into chronic pain, shifting from a physiologic to a pathological type of pain in a process sometimes called “wind-up” or “chronification” [13]. While chronic pain is characterized by centralization, there has not emerged a clear causative link that would cause acute pain to transition to another mechanism entirely. In this connection, it is important to note that acute postoperative pain does not transition to chronic pain in most patients and optimal control of postsurgical pain does not always prevent CPSP [8,10]. Nerve lesions and hyperalgesia secondary to surgery may be the important contributing factors to CPSP [14].

While the trajectory of postoperative pain is well-defined for many procedures, acute postoperative pain can be complex, dynamic, and changeable. Many studies of CPSP rely on pain intensity scores as the sole metric of evaluation for postoperative pain, which are inadequate to capture the multiple dimensions of pain. Pre-existing pain syndromes may result in more prolonged periods of postoperative pain; chronic pain patients using opioid analgesia before surgery may experience a longer pain trajectory following surgery [15]. A study of 164 hip or knee arthroplasty patients found that symptomatic improvement occurred in the first three postsurgical months with little change in months three to 12 [16]. An intriguing new paradigm of postsurgical pain trajectories divides surgical patients into three main groups. The first and by far the largest group (57%) experiences little pain on the first day after surgery with an unproblematic resolution of painful symptoms over the next four days [17]. The next-largest group of patients (30%) experiences severe pain immediately following surgery but has rapid pain resolution over the next few days. The smallest group (13%) has the most problematic experience, i.e., severe pain right after surgery that does not resolve over the next few postoperative days. This last group is more likely to have higher pain intensity scores six months after surgery than the other two groups of patients. This suggests that postsurgical trajectories vary among patients and that effective pain resolution over the immediate few days after surgery may be a more accurate predictor of CPSP than the severity of pain immediately after surgery [17].

This exposes a very important clinical consideration, in that most surgical inpatients are discharged while still experiencing acute postoperative pain. Despite discharge instructions, it is not known how well these patients adhere to their pharmacological advice. Patients with CPSP have undergone a transitional phase of up to three months where so-called transitional pain is experienced [10,18]. Transitional pain is not well understood and represents a “gray zone” that is typically not treated in a clinical setting. In fact, there is little information about the nature and evolution of transitional pain. Yet, this transitional pain may be foundational to a better elucidation of CPSP. At a minimum, transitional pain may help to elucidate the known phenomenon of pain chronification. It has been hypothesized that effective pain control during this transitional phase may help to mitigate or even prevent CPSP.

In a study of 279 thoracic surgery patients followed for a year after surgery, three distinct postsurgical trajectories emerged. The largest group experienced mild pain over the course of the first 12 months after surgery. The other two groups were smaller and similar in size. The second group suffered moderate pain immediately following the operation which decreased substantially by three months and was mild at 12 months. The third group had moderate pain immediately after surgery which persisted over the course of the next 12 months [19]. Pain levels following surgery and levels of pain catastrophizing at baseline were predictors of which pain trajectory the patient would follow, but surgical variables or type of anesthesia were not predictive [19].

An observational study of 87 orthopedic surgery patients, with a mean age of 62.4 years, followed patients from baseline to one year after surgery [20]. Postsurgical pain affected 97.4% of patients at 10 days, 81.2% at six weeks, and 79.5% at three months. At 12 months after surgery, surgical site pain was still present in 65.5% of patients and 29.9% reported moderate to severe incidental pain. This study suggests that pain intensity at 30 days correlates with pain intensity at three and six months, implying that better pain management in this early postoperative phase would be beneficial. Further, in this study, subacute pain at 30 days after surgery predicted CPSP at 12 months. At three months, moderate to severe pain with a neuropathic component, as measured on the Doleur Neuropathique (DN) questionnaire, found that a score of ≥3 correlated with CPSP at one year [20]. Results from this study may not be generalizable because the study had no control group and enrolled only patients undergoing orthopedic surgery.

The question naturally arises as to how can we better manage transitional pain and reduce the risk of CPSP. A recent meta-analysis has provided evidence that local anesthetics and regional anesthesia might reduce the risk of CPSP better than conventional analgesic approaches. This systematic review included 41 studies with a total of 3,143 patients [21]. The meta-analysis found moderate evidence that thoracic epidural anesthesia and regional anesthesia prevented CPSP in 25% of patients. There was low-quality evidence that paravertebral block and local infiltration agents reduced CPSP [21]. Evidence in support of regional or local anesthesia to prevent CPSP is in its early stages and remains equivocal [22] (Table 2).

**Table 2:** Regional anesthesia and/or local analgesic techniques with evidence supporting their role in reducing or preventing CPSP based on the type of surgery. CPSP: chronic postsurgical pain; IV: intravenous; LA: local analgesia; PVB: paravertebral block; RA: regional anesthesia; TAP: transabdominal plane block

Surgery	RA or LA techniques
Breast surgery	IV lidocaine, continuous wound infusion, lidocaine cream PVB
Thoracic surgery	Epidural PVB
Cesarean section	Intraperitoneal installation, TAP block, continuous wound infusion
Iliac bone graft	Continuous wound infusion, multiple wound infiltrations, local wound infiltration
Amputation	Epidural
Cardiac surgery	Lidocaine patch
Laparotomy	Epidural
Inguinal hernia repair	Local wound infiltration

Treatments once reserved for chronic pain are being explored for perioperative use with the goal that they might prevent CPSP. Such techniques include radiofrequency ablation, cryoneurolysis, and neuromodulation, which can be combined with nerve blocks or other conventional acute pain management techniques. This applies to pharmacologic treatments as well, where agents for long-term pain control, such as gabapentinoids or selective serotonin reuptake inhibitors, may sometimes be administered for perioperative pain management [23].

There is considerable variation in regional analgesic approaches. For example, regional analgesia for acute pain management can be short-lived, such as local anesthetic-based peripheral nerve blocks, or long-lasting, such as percutaneous cyroneurolysis or percutaneous peripheral nerve stimulation [24]. Cryoneurolysis uses low temperatures to temporarily and reversibly ablate a peripheral nerve; its effects may last weeks or months. Percutaneous peripheral nerve stimulation uses an insulated lead adjacent to the peripheral nerve and sends an electric current to that lead via an external pulse generator; it can be used for 60 days [25]. In a clinical study of 66 orthopedic outpatients, it was found that patients who received percutaneous peripheral nerve stimulation consumed on average 5 mg of opioids per day compared to 48 mg per day among the control patients who received sham neuromodulation treatment. Moreover, the sham patients had a mean pain score of 3.1 ± 1.7 compared to 1.1 ± 1.1 in the neuromodulation group [25]. A limiting concern for such postoperative interventions can be cost, but evidence suggests that such procedures can be cost-effective in the population of patients at elevated risk for CPSP [26-28].

The lack of continuity of care may also play a role in CPSP because perioperative pain management in most clinics is fragmented by definition. Most hospitals separate acute from chronic pain services as isolated entities with minimal interaction [29]. Perioperative and postoperative pain care is often discontinued when the patient is discharged. Pain arising in the immediate postoperative period is often managed by the primary surgeon, who is rarely trained in postoperative analgesia. Perioperative nerve injury is not uncommon but may resolve on its own and lead to the notion that postoperative pain will resolve with time too [30]. Opioids often become a default pain management solution because of their familiarity and ease of prescribing, but they are not always the optimal analgesic.

Unfortunately, the current paradigm is to manage postoperative pain in a reactive and conventional model with an emphasis on temporary solutions rather than a more evidence-based and comprehensive approach [31]. For improved management of postsurgical pain and prevention of acute postsurgical pain transitioning into chronic postsurgical pain, the establishment of chronic postsurgical pain services should be emphasized [32,33]. Such a department would have several goals. These goals would include tapering and/or deprescribing opioid analgesics, recommending pre- and post-discharge interventions for pain, prescribing postoperative analgesics as needed, offering educational interventions and/or behavioral therapies, working with the patient to improve function, alleviating distress, and optimizing outcomes, as well as, finally, working with the healthcare system to reduce lengths of stay, costs, and healthcare resource utilization.

There is an urgent and unmet medical need to better understand CPSP, to better quantify its risks, to manage surgical patients with the goal of preventing CPSP when possible, and to educate clinicians and their patients. Transitional pain warrants further study. CPSP is not a sign of surgery gone but it may be an indication that postoperative pain management was insufficient.

Knee Osteoarthritis

The potentially debilitating pain associated with knee osteoarthritis affects over nine million Americans and is the most common cause of knee pain globally [34]. Shifting demographics and the “graying” of the developed world are likely to lead to substantial increases in cases of knee osteoarthritis. Treatments range from nonpharmacologic interventions, such as exercise, to drug therapy, injections, and interventional care including total knee replacement [35,36]. Multimodal approaches, whether in diagnostic strategies or analgesia, can be helpful but require patient education and commitment [37,38].

The pain associated with knee osteoarthritis may be treated first-line with non-steroidal anti-inflammatory drugs (NSAIDs), such as dexketoprofen or ibuprofen. Mepivacaine or ropivacaine may be injected intra-articularly [39]. Methotrexate and ozone therapy are also described in the literature [40]. Hyaluronic acid injections (viscosupplementation) and platelet-rich plasma injections are being evaluated [41]. Intra-articular injections may be guided by landmarks, fluoroscopy, or ultrasound, with excellent evidence available for ultrasound-guided procedures. Fluoroscopy exposed patients to ionizing radiation should be avoided if a non-radiant treatment option is available.

Viscosupplementation using hyaluronic acid has a modest efficacy but a high response rate; it offers about 20% efficacy over placebo in treating knee osteoarthritis pain, but more than half (approximately 60%) of patients respond to the treatment, which is also well tolerated [42]. While cartilage protection is not yet confirmed, viscosupplementation can be opioid-sparing and delay joint replacement. Results commence after one to four weeks but are durable for six months or more [42]. Injections should not be administered when synovitis or effusion is detected because injection in the presence of synovitis can result in irritation and trigger symptoms of acute arthritis, while effusion can dilute the injectate, making it less efficacious [42]. Avoid injecting into a background of crystal arthropathies, which can indicate gout or gouty arthritis [43]. There may be a slight decrease in cartilage thickness with each injection, so clinicians may wish to minimize the use of repeated injections [44]. Viscosupplementation gives synovial fluid the opportunity to behave differently based on load, in other words, when joint stress is low, hyaluronans are viscous but when joint stress increases, hyaluronans are more elastic and absorb energy more efficiently. This variability can benefit patients with knee osteoarthritis [45].

Chondroprotective substances preserve mitochondrial function and improve mitochondria-modulated apoptosis [46]. They can suppress pro-inflammatory cytokines in the joint and relieve pain by reducing the knee joint inflammation which can make nociceptors excitable and increase proprioception. Quarterly injections of hyaluronic acid into the knee of patients afflicted with knee osteoarthritis provide rapid, safe, and effective analgesia in older patients with mild to moderate knee osteoarthritis over five years [47]. A meta-analysis confirmed a key benefit of hyaluronic acid injections is safety and tolerability [48]. In a head-to-head study of short-term outcomes using hyaluronic acid injections versus oral NSAIDs in treating the pain associated with knee osteoarthritis, hyaluronic acid injections offered statistically significantly greater pain reduction and improved function, but these statistical benefits did not rise to the level of clinically important. There was an overall lower risk of adverse events with hyaluronic acid compared to oral NSAIDs as well [49].

Platelet-rich plasma can contain a mixture of both anabolic and catabolic mediators but may confer analgesic benefits to patients with knee osteoarthritis [50]. When injected into synovial joints with non-chondrocytic cell lineages, for instance, fibroblast-like synoviocytes, this can result in the production of matrix metalloproteinases (MMPs) that mediate catabolism of cartilage. Thus, synoviocytes exposed to platelet-rich plasma secrete MMPs, resulting in increased catabolism of the cartilage, which can launch a pro-inflammatory response [50].

Intra-articular corticosteroids were shown in a systematic review and meta-analysis to be more beneficial than placebo in terms of reducing pain and improving function in individuals with osteoarthritis of the knee. Injections decreased pain intensity with effects diminishing over time and lasting less than six months [35].

Comparative studies have evaluated different analgesic injections for knee osteoarthritis. A meta-analysis of five randomized clinical trials (n = 1,004 patients total) compared the safety and analgesic efficacy of intra-articular hyaluronic acid compared to intra-articular methylprednisolone in patients with knee osteoarthritis pain [51]. The study found both therapies were safe and effective without any significant differences in long-term follow-up of adverse effects. Pain, function, and stiffness were evaluated using the Western Ontario and McMaster University Osteoarthritis Index (WOMAC) metric at four, 12, and 26 weeks [51]. In a systematic review comparing platelet-rich plasma to hyaluronic acid for knee pain due to osteoarthritis, 12 studies were used and showed that while clinical outcomes were similar between groups at six and 12 months, platelet-rich plasma provided significantly greater analgesic benefit at six and 12 months [52]. Comparing platelet-rich plasma to hyaluronic acid for knee osteoarthritis in a systematic review of 18 studies, mean improvement was significantly greater among platelet-rich plasma patients (44.7%) than hyaluronic acid patients (12.6%) when measured on the WOMAC scale [53]. Platelet-rich plasma groups also had significantly less intense pain. Interestingly, it was noted that leukocyte-poor platelet-rich plasma may be more beneficial than leukocyte-rich plasma according to International Knee Documentation Committee scales [53].

Platelet-rich plasma and hyaluronic acid can be used for intra-articular injections either alone or in combination, and the combination offers better WOMAC function and total scores at six months as well as improved scores on the Lequesne index. The combination of platelet-rich plasma with hyaluronic acid has similar side effects as either agent used alone, in other words, combination therapy does not increase side effects [54].

Sensory and sympathetic neurotransmitters play a key role in joint tissue and bone homeostasis, modulating articular cartilage, subchondral bone, and synovial joints. Changes in peripheral joint innervation may lead to degeneration of joints, leading to osteoarthritis [55]. It may be argued that osteoarthritis is not just degeneration of articular cartilage but rather involves the failure of the entire joint as an organ, encompassing disorders of the bone, ligaments, synovium, and joint capsule itself [56]. In fact, osteoarthritis of the knee is increasingly presented and studied as a disease of the entire knee joint [57].

Botulinum toxin is a potent neurotoxin with an affinity for the cholinergic synapse, thus blocking the release of acetylcholine, which is being investigated for a potential role in certain pain syndromes, including osteoarthritis [58]. In this regard, it is speculated that botulinum toxin may indirectly affect the central nervous system [58]. Botulinum toxin dosage is based on the patient’s weight and results are durable for six to 12 months [59]. A meta-analysis of five randomized clinical trials found that botulinum toxin type A was significantly better in reducing pain caused by knee osteoarthritis on both a visual analog pain scale and the WOMAC questionnaire for use over the short or long term, defined as ≤4 or ≥8 weeks, respectively [60].

Ultrasound and other imaging technologies have advanced interventional pain medicine. An ultrasound-guided genicular nerve block can be combined with a local anesthetic and corticosteroid, which may offer short-term analgesia, but the addition of the corticosteroid does not offer an incremental benefit over the use of the local anesthetic alone [61]. A case series in the literature offered a technique for patients with pain from knee osteoarthritis who were neither candidates for joint replacement nor successful in conservative pain management [62]. The series treated three patients with thermal radiofrequency ablation, which reduced their WOMAC score by 54% at four weeks. Two patients underwent a chemical neurolysis procedure which reduced WOMAC scores by 49% at four weeks. Using neural ablation with a modified landmark approach may be an appropriate treatment for this particular subpopulation of knee osteoarthritis patients [62].

Thoracic Trauma

Chest trauma with its multiple presentations is a significant source of morbidity and mortality, both for direct effects of the injury itself as well as potentially life-threatening secondary complications, such as respiratory failure [63]. The most common form of thoracic trauma is rib fracture, which involves sufficient energy to damage tissue and cause pulmonary contusions. Because of the densely innervated intercostal region, rib fractures can cause intense pain, which can impede mobility and rehabilitation [63]. Pain can impair the patient’s ability to cough and clear the lungs adequately, may limit deep breathing, cause retention of sputum leading to atelectasis and respiratory infections and distress, can reduce tidal volumes, and may decrease lung compliance. Pain becomes the focal point in the vicious cycle of thoracic complications following chest trauma.

In an observational study, 54 patients who suffered isolated chest trauma the previous year were surveyed about their injuries and the impact these injuries had on their lives. The majority of these patients (79.6%) had chronic pain with a median intensity of 3.18 ± 1.4 on an 11-point scale and 90.7% had neuropathic pain [64]. Treating this pain is imperative; the ideal analgesic strategy would be simple, safe, durable, efficient, and have minimal side effects. This treatment would help improve respiratory dynamics, clear secretions, allow for early mobilization and rehabilitation, and permit chest physiotherapy. Over the years, many approaches have been used. Systemic opioid analgesics are associated with sedation and potentially treatment-limiting side effects. Thoracic epidural anesthesia is likely associated with safety issues, such as vascular puncture and possible respiratory depression. More recently, nerve blocks have been evaluated as offering adequate analgesia with enhanced tolerability. Myofascial plane blocks utilize a simple technique for administration and they offer unilateral analgesia, multilevel coverage, and a favorable profile in terms of safety and side effects.

**Figure 1:**
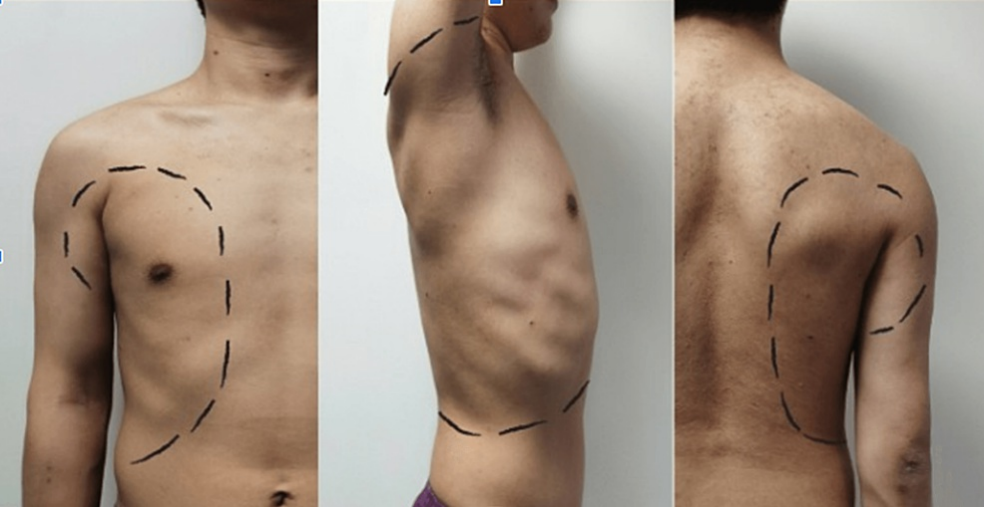
For an ultrasound-guided serratus anterior plane blockade, T2-T6 are the levels for the anterior chest wall, T2-T8 for the lateral chest wall, and T2-T9 for the posterior chest wall. The photograph is provided by the authors and used with the permission of the patient.

Several different regional techniques are available, but the serratus anterior plane (SAP) and erector spinae plane (ESP) blocks are of particular interest. In 2013, Blanco and colleagues published their ultrasound-guided SAP technique for analgesia following surgery on the hemithorax [65]. This approach has been used to effectively manage the pain associated with rib fractures [65]. The nerve roots of the thoracic spine split into ventral and dorsal rami. The dorsal ramus leads to the intercostal nerve, which has anterior and lateral posterior branches. The anterior serratus muscles are located on the sides of the chest wall, from the anterior of the first through ninth ribs; the serratus muscles terminate at the medial border to the scapula. The intracostal nerve innervates this area by traveling through the spaces between the muscles. The SAP blockade targets the lateral cutaneous branch of the intracostal nerve, providing analgesia to approximately two-thirds of the anterior region, making it suitable for pain control at the hemithorax [66]. The skin area is depicted in Figure 1.

The SAP block may be administered with the patient in a sitting or lateral position. The ultrasound transducer is positioned around the parasagittal at the mid-axillary line at the level of the fifth rib. The injection may be superficial or deep into the serratus anterior muscle or both [66]. Caution is advised with respect to the thoracodorsal artery, which originates in the axillary region and runs across the lateral edge of the scapula near the thoracodorsal nerve in the superficial fascia between the muscles [67].

Studies comparing SAP block with other techniques have found SAP to offer safe and effective analgesic benefits with hemodynamic stability [68]. Another study found safe and effective pain control with opioid-sparing effects and shorter lengths of stay in the hospital [69]. A meta-analysis of seven randomized controlled trials (n = 489 total) found SAP blockade had a significant opioid-sparing effect and decreased the rate of postoperative nausea and vomiting in patients undergoing video-assisted thoracoscopic surgery (VATS) [70]. In a study of patients undergoing elective VATS lung resection, postoperative respiratory function as well as pain control was significantly better in the SAP group, who also had lower rates of pneumonia, pulmonary atelectasis, hypoxemia, and vomiting [71].

ESP block is a novel type of sensory block that may be of value in treating chronic neuropathic pain and acute pain that might occur as a result of rib fracture or certain types of chest surgery. It has been proposed as a potential alternative to other central neuraxial techniques; compared to these other approaches, ESP block is technically easier and safer to perform [72]. The erector spinae consists of three large muscle groups, namely, the iliocostalis, the longissimus, and the spinalis, which run bilaterally down the spine from the skull base to the sacral region. These muscle groups extend from the spinous to the transverse processes, extending outward to the ribs. ESP block can cover the anterior, lateral, and posterior areas of the thoracic wall [73]. An observational study of 12 healthy volunteers who underwent ultrasound-guided ESP block with 0.5% ropivacaine 20 mL at the left T6 transverse process were shown to have a loss of cold sensor perception primarily from T4 to T11 with the most pronounced effect at T6 to T9. However, the effect did not extend to the anterior chest, lateral chest, or walls of the abdomen, suggesting that the dorsal branch of the spinal nerve was blocked [74].

ESP block requires that the patient be sitting or prone on the side; most patients prefer to be seated. Under ultrasound guidance, the transducer is placed on the skin in a paramedian sagittal position approximately 2 cm from the midline. This allows visualization of the ribs, which appear like acoustic shadows on the ultrasound. As the transducer is advanced medially, the rib-transverse complex will come into view as a flat, gray, square-shaped hyperechoic. The pleura should not be visible [75].

ESP has a short-term opioid-sparing effect, even in geriatric surgery patients [76-78]. A meta-analysis of breast cancer surgery patients (n = 679 in 11 randomized controlled trials) found that ultrasound-guided ESP provided effective analgesia and reduced morphine consumption in the first 24 hours after surgery compared with general anesthesia alone [79]. The method is being widely adopted around the world and provides effective pain control although the mechanisms of action behind it are not yet elucidated.

Facet Joint Arthropathy

Axial back pain has many potential causes, with lumbar facet arthropathy a common etiology. Facet joint pain is prevalent, particularly among geriatric patients, but has not been the subject of as much research interest as knee osteoarthritis pain [80]. Facet joints are innervated where the spinal nerve bifurcates into an anterior and a posterior primary ramus. The posterior primary ramus divides into lateral, intermediate, and medial branches. The medial branches are the source of innervation to the facet joint, with each facet joint served by two adjacent branches, one at the same level as the facet joint and the other from the level above it. Medial nerve branches take their names from the somatic nerve root from which they originate. Thus, each facet is a three-joint unit and the movement or alignment of any one affects the other two. Degenerative disc disease often precedes facet arthritis. When two vertebrae are fused, it can accelerate degeneration in the adjacent levels.

Like other interventional pain procedures, successful arthropathy is predicated on an accurate diagnosis, proper identification of the pain generator, selection of appropriate surgical candidates, and precise identification of interventional targets. Multiple pain mechanisms may be present in an individual patient, and different pain generators may be at play in one patient. It is important to confirm that the needle has hit the right target and is in the proper position before injecting dye. Risk factors for facet arthroplasty include older age, repetitive motion, low-grade trauma, and any acute event that stretches the joint beyond its limit, also known as whiplash.

Facet arthrosis was present in 53% (L1-L2), 66% (L2-L3), 72% (L3-L4), 79% (L4-L5), and 59% (L5-S1) of cadaveric lumbar spines in an anatomic study [81]. By age, facet arthrosis was present in 57% of those aged 20 to 29 years, 82% of those aged 30 to 39 years, and >90% of those aged >40 years [81]. In this study, 100% of all individuals over the age of 60 years had facet arthrosis. Men are more likely than women to have facet arthrosis, and it is more likely to be severe in men at all lumbar levels [81]. Despite the association of certain rheumatic conditions with obesity, facet joint arthrosis appears to be independent of body mass index [82].

Facet joint arthropathy is exceedingly challenging to diagnose accurately and, for that reason, it is not always properly identified and thus may go untreated. Furthermore, even when appropriately diagnosed, facet joint arthropathy is not well understood by clinicians [83]. In taking a history from a patient with degenerative disc disease, one key symptom suggestive of facet joint arthropathy is axial back pain of more than six weeks duration. In some cases, pain can be referred to the groin or thigh, but rarely below the knee. All red flags must be identified and ruled out. Computed tomography and other imaging techniques are not helpful in making such diagnostic determinations in zygapophysial joints [84]. However, caution is urged as even a complete physical examination and patient history may be insufficient for an accurate diagnosis. The gold standard in facet joint arthropathy diagnosis is a positive response to medial branch block, defined as ≥50% reduction in pain when lidocaine is injected around the area of the medial branch. Such a positive response can be followed by radiofrequency ablation of the medial branches for analgesic relief that can last six to 12 months [85].

Facet joint injections require practical as well as medical and technical expertise. Ultrasound is preferred over fluoroscopy due to radiation concerns and the ability to visualize blood vessels on ultrasound. The ultrasound equipment should be set up in such a way that the monitor remains in the clinician’s line of sight at all times. The clinician should consider ergonomics in terms of position; whether sitting or standing, the clinician should have some support for the arm holding the needle. The clinician should hold the needle in the dominant hand, particularly if they are less experienced in this technique, and the probe in the other. The patient’s head should not be permitted to turn >45 degrees and the torso should be prevented from arching [86].

When performing a medial branch block, use minimal amounts of local anesthetics for the skin and muscle and a smaller-diameter needle in the range of 25G/22G. Avoid the use of sedation. Contrast dye can be used to rule out vascular placement, but the needle position should be double-checked when using dye, and it is important to be aware that the contrast media may not spread out as anticipated. Do not use steroids during a facet joint medial branch block. The success of the block can be assessed by the patient’s response. A successful diagnostic lumbar facet joint nerve block should provide immediate or near-immediate 75% to 100% pain relief.

Discussion

The purpose of this session at the Tunis pain conference was to address four familiar but highly challenging pain scenarios and the latest treatment techniques and goals. CPSP is a prevalent condition, but it is not entirely clear why surgical pain transitions to chronic and centralized pain, often with a dominant neuropathic component, in some patients but not others. This sheds light on a little-studied subject of “transitional pain” or pain in a subacute setting. It may be that learning more about transitional pain will shed light on the process of wind-up or chronification [87]. Advances in treatment include more aggressive treatment of acute postsurgical pain, possibly involving percutaneous neuromodulation [17].

Knee osteoarthritis is also a very common form of pain and dysfunction, for which treatments currently seem to be pain management followed by joint replacement. Viewing knee osteoarthritis holistically as a disease of the knee joint, intra-articular injections may be appropriate, and head-to-head studies offer clinical guidance [88]. Platelet-rich plasma, hyaluronic acid, and botulinum toxin are being investigated in this regard

Chest trauma can be associated with intense pain and morbidity, but it is often treated with pharmacologic means and watchful waiting. The role of the regional blockade is to allow for better and safer pain control in these patients [89].

Finally, facet joint arthropathy can be a source of pain, particularly in older patients, for which treatment approaches vary from pharmacological therapy to various types of surgery. Ultrasound-guided regional blocks can provide safe, effective pain control [90]. It is interesting that facet joint arthropathy is both very prevalent and not the subject of intense research.

General practitioners, emergency and trauma specialists, as well as pain physicians treat these types of patients frequently. In each syndrome, it is important to consider the patient as an individual. Some interventional techniques are offering safer, longer-lasting, and more diverse treatment options.

## Conclusions

From ancient to modern times, physicians have been challenged by treating pain, and four particular pain syndromes have emerged at the recent conference in Tunis as being of particular interest, namely, CPSP, knee osteoarthritis, chest trauma, and facet joint arthropathy. CPSP is a prevalent condition that is being discussed today in terms of interrupting the transition from expected and adaptive acute postsurgical pain to chronic pain. Knee osteoarthritis is prevalent and likely to increase with the aging population. New research is focusing on chondroprotective substances aimed at preserving mitochondrial function. Platelet-rich plasma may also confer relief for those with knee osteoarthritis. Thoracic injuries, e.g., those occurring in vehicular accidents, may benefit from regional techniques, including ESP. Facet joint arthropathy is a common reason for chronic back pain. Getting an accurate diagnosis and identification of joints can be a challenge but may be effectively treated with ultrasound-guided injections. Further study in these four common but challenging pain syndromes is warranted as is greater awareness of these conditions by clinicians.
